# Food safety impacts of antimicrobial use and their residues in aquaculture

**DOI:** 10.1186/s40985-018-0099-2

**Published:** 2018-08-08

**Authors:** Reuben Chukwuka Okocha, Isaac Olufemi Olatoye, Olufemi Bolarinwa Adedeji

**Affiliations:** 1 Department of Veterinary Public Health and Preventive Medicine, University of Ibadan, Ibadan, Nigeria; 2 Paul Allen G. School for Global Animal Health, Washington State University, Pullman WA, USA

**Keywords:** Antimicrobial residue, Antibiotic resistance, Public health, Aquaculture and food safety

## Abstract

**Background:**

Residues of antimicrobials in food have received much attention in recent years because of growing food safety and public health concerns. Their presence in food of animal origin constitutes socioeconomic challenges in international trade in animal and animal products. The major public health significances of antimicrobial residues include the development of antimicrobial drug resistance, hypersensitivity reaction, carcinogenicity, mutagenicity, teratogenicity, bone marrow depression, and disruption of normal intestinal flora. Indiscriminate use of antimicrobials in aquaculture resulting in occurrence of residues in aquaculture products and associated harmful health effects in humans requires control measures to ensure consumer protection.

**Main body:**

This article focuses on factors contributing to the presence of antimicrobial residues in aquaculture products and their implications on consumers’ safety. Regulatory actions aimed at prudent use of veterinary drugs in food-producing animals with emphasis on aquaculture for safe and wholesome food production are also reviewed.

**Conclusion:**

Prudent use of antibiotics in aquaculture under veterinary supervision is critical in ensuring safety of aquaculture products. Good animal husbandry practices as well as the use of alternatives to antibiotics such as vaccination, probiotics, phage therapy, and essential oils are recommended panaceas to reducing the use of antimicrobial residues in aquaculture and consequent food safety effects.

## Background

Aquaculture continues to be the fastest growing animal food-producing sector, accounting for about 46% of total food fish supply to meet the protein needs of the increasing world population [1]. China, India, Vietnam, Bangladesh, and Egypt engage in more aquaculture than wild-caught fish. There has been a general increase by all continents in aquaculture share of total fish production, but in Oceania this share has been declining [2]. China has been reported to contribute more than 60% of the global aquaculture production and also administers large amounts of antibiotics to ensure adequate productivity and disease management [3].

The current intensification of aquaculture has led to the promotion of conditions that favor the development of infection and disease-related problems and biofouling. Consequently, antimicrobial regimens are being employed prophylactically and therapeutically to manage these diseases as well as to enhance growth promotion. In aquaculture, antibiotics are generally administered in surface-coated or pelleted feeds or via other routes such as water immersion or by injection.

The global antimicrobial use in food animals including aquaculture is increasing tremendously, estimated at 63,151 tons in 2010, and projected to rise by 67% in 2030. Brazil, Russia, India, and South Africa have the highest estimated global antimicrobial consumption [4]. Antibiotics have not always been used responsibly in aquaculture, and control of their use has not provided proper assurance of the prevention of risks to humans [5]. Responsible use of antibiotics requires clear instructions from drug manufacturers, proper handling and distribution by dealers, and veterinary supervision of administration by farmers with observance of withdrawal periods before slaughter.

Unregulated use of antibiotics in aquaculture industry for the production of farm-raised fish and shrimps could pose human health and food safety concerns that remain largely unaddressed in most developing nations of the world. A consequence of the use of the antibiotics in food-producing animals is the presence of drug residues, even in very low concentrations, in the edible tissues of the treated animal. Antimicrobials used according to label directions should not result in residues at slaughter. However, reasons for residue presence in edible tissues include non adherence to recommended label directions or dosage (extra-label usage); non observance of recommended withdrawal periods; administration of too large a volume at a single injection site; use of antibiotic-contaminated equipment, or failure to properly clean equipment used to mix or administer drugs; mixing errors; unintentional feeding with spilled chemicals or medicated feeds; animal effects, such as age, pregnancy, congenital, illness, and allergies; chemical interactions between drugs; variations in water temperature for aquatic species; environmental contamination; and improper use of drugs [6]. The presence of antibiotic residues in aquaculture products could result in the development of bacterial resistance and toxicity to consumers that can lead to morbidity and/or death. Chloramphenicol residues, for example, lead to an increased risk of developing cancer and in very low concentrations may trigger aplastic anemia, a disease that causes bone marrow to stop producing red and white blood cells and is often irreversible and fatal. Other toxic effects include immunopathological effects and carcinogenicity by sulphamethazine, oxytetracycline, and furazolidone; mutagenicity and nephropathy by gentamicin; and allergy by penicillin [7].

The presence of antibiotic residues in domestic animal products and the associated consumers’ health hazards have been reported with little attention focused on the aquaculture industry. This article reviews the pattern of antibiotic use in aquaculture and the food safety consequence associated with residues in aquaculture products as well as legislation on drug use, public health implications, and basic principles for prudent and rational use of antimicrobials in aquaculture.

## Main text

### Antimicrobial agents

Antimicrobials have been used for inhibiting the growth or multiplication of a wide range of bacteria in human and veterinary medicine since the discovery of penicillin by Alexander Fleming in 1929 [8]. Subsequent development by Ernst Chain and Howard Florey during the World War II led to the antimicrobial revolution, which has been followed by the development of many other classes of antimicrobials [7, 9]. Today, antimicrobials play a major role in modern livestock production for prevention and treatment of diseases as well as growth promotion. Administration of antibiotics in food animals has been unbridled in many countries due to weak regulations, poor management practices, and disease endemicity [10, 11]. The global rise in production and demand for aquaculture products has resulted in increasing dependence on antibiotics with resultant residues in the products produced for human consumption.

### Antimicrobial residues

Irrespective of the route or purpose of administration, antimicrobials can accumulate as residues in tissues, before they are completely metabolized or excreted from the body. The occurrence of residues in fish or other animal tissues is most likely when animals are harvested for human consumption while still on medication or shortly after medication before the withdrawal period elapses [12]. Consumption of such products may result in many health problems in humans [13, 14]. Chiefly among the health concerns is the development and propagation of antimicrobial resistance along the food chain.

Antimicrobial residues can also occur in fish when the drugs are administered outside the labeled dose or recommendations [15]. Extra-label usage of antimicrobials in aquaculture is practiced but supposed to be supervised by veterinarians. Chloramphenicol, dimetridazole, ipronidazole, nitroimidazoles, furazolidone, nitrofurazone, and fluoroquinolones are prohibited for extra-label use in food-producing animals [16].

Food safety efforts and monitoring are required by international standards set by the joint Food and Agriculture Organization of the United Nations and the World Health Organization (FAO/WHO) Codex Alimentarius Commission. Maximum residue limits (MRLs) of approved veterinary drugs in food are set with legally permitted quantities of parent drugs and/or metabolites in food products of treated animals that are safe for consumers (National Council regulation EEC/2377/90 [17]). Although efforts have been made to harmonize maximum residue limits worldwide under the aegis of World Trade Organization (WTO) and the Codex Alimentarius, MRLs still vary from one geographical location to another. In fact, MRLs in a particular animal product may differ from one country to another depending on the local food safety regulatory agencies and drug usage patterns [18] and most developing countries have yet to develop their own MRLs. The MRL values for antibiotics in fish according to European Union legislation are presented in Table 1 [19].

**Table 1:** MRL values for antibiotics in fish according to European Union legislation: Commission Regulation (EU) No 37/2010 of 22 December 2009 [19]

Pharmacologically active substance	Marker residue	MRL (μg kg^−1^)^a^
Sulfonamides (All substances belonging to the sulfonamide group)	Parent drug	100^b^
Diaminopyrimidine derivatives Trimethoprims	Parent drug	50
Penicillins		
Amoxicillin	Amoxicillin	50
Benzylpenicillin	Benzylpenicillin	50
Cloxacillin	Cloxacillin	300
Dicloxacillin	Dicloxacillin	300
Oxacillin	Oxacillin	300
Quinolones		
Oxolinic acid	Oxolinic acid	100
Danofloxacin	Danofloxacin	100
Difloxacin	Difloxacin	300
Enrofloxacin	Sum of enrofloxacin and ciprofloxacin	100
Flumequine	Flumequine	600
		150 (salmonidae)
Sarafloxacin	Sarafloxacin	30 (salmonidae)
Macrolides		
Erythromycin	Erythromycin A	200
Tilmicosin	Tilmicosin	50
Tylosin	Tylosin A	100
Florfenicol and related compounds		
Florfenicol	Florfenicol	1000
Thiamphenicol	Thiamphenicol	50
Tetracyclines		
Chlortetracycline	Sum of parent drug and its 4-epimer	100
Oxytetracycline	Sum of parent drug and its 4-epimer	100
Tetracycline	Sum of parent drug and its 4-epimer	
Lincosamides		
Lincomycin	Lincomycin	100
Aminoglycosides		
Spectinomycin	Spectinomycin	300
Neomycin (including framycetin)	Neomycin B	500
Paramomycin	Paramomycin	500
Polymyxins		
Colistin	Colistin	150
Nitrofurans		No maximum levels can be fixed

Source: Cañada-Cañada et al. 2009 [14]

^a^For fin fish, these MRLs relate to “muscle and skin in natural proportions”

^b^Combined total residues for all substances within the sulfonamide group should not exceed 100 μg kg^−1^

Acceptable daily intake (ADI) is also a critical standard set from toxicological studies based on No Observable Effect Level (NOEL) and safety factor (often 100) [20]. ADI is an estimate of the residue that can be ingested daily over a lifetime without a health risk to the consumer.

**Fig. 1:**
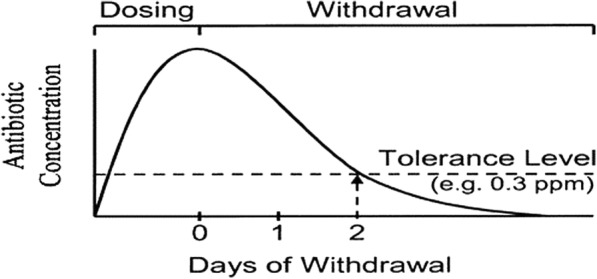
Theoretical representation of withdrawal period. Adapted from: Donoghue [25]

At the farm level, withdrawal periods are indicated for drugs used in different species of animals as the period of time post-administration of such drugs that must elapse before the edible products are considered safe (i.e., when the residue levels are below the MRLs). Theoretical representation of tolerance level and withdrawal period is shown in Fig. 1. Withdrawal periods are set by drug manufacturers during which time treated animal products are not supposed to enter the food chain. When extra-label use of antimicrobials is allowed by veterinarians, it is expected that the withdrawal period should be adjusted accordingly with most times extended to minimize the chances of accumulation of the residues in animal tissues [21]. The withdrawal periods of some antimicrobials used in fish are presented in Table 2.

**Table 2:** Withdrawal periods of some antibiotics in yellowtail, rainbow trout, and kuruma prawn

Antibiotic	Target species	Administration mode	Withdrawal period (days)
Amoxicillin	Yellowtail	Oral	5
Ampicillin	Yellowtail	Oral	5
Erythromycin	Yellowtail	Oral	30
Oxytetracycline	Yellowtail	Oral	20
Oxolinic acid	Yellowtail	Oral	16
Spiramycin	Yellowtail	Oral	30
Novobiocin	Yellowtail	Oral	15
Flumequine	Yellowtail	Oral	–
Lincomycin hydrochloride	Yellowtail	Oral	10
Florfenicol	Yellowtail	Oral	5
Thiamphenicol	Yellowtail	Oral	15
Oxytetracycline hydrochloride	Rainbow trout	Oral	30
Oxolinic acid	Rainbow trout	Oral	21
Sulfadimethoxine	Rainbow trout	Oral	30
Sulfamonomethoxine	Rainbow trout	Immersion	15
Florfenicol	Rainbow trout	Oral	14
Oxytetracycline hydrochloride	Kuruma prawn	Oral	25
Oxolinic acid	Kuruma prawn	Oral	30

Source: Southeast Asian Fisheries Development Center, Aquaculture Department [96]

## Use of antimicrobials in aquaculture

In addition to their use in human medicine, antimicrobials are also used in food animals and aquaculture, and their use can be categorized as therapeutic, prophylactic, or metaphylactic. Therapeutic use corresponds to the treatment of established infections. Metaphylaxis is a term used for group-medication procedures that aim to treat sick animals while also medicating others in the group to prevent disease. Prophylaxis means the preventative use of antimicrobials in either individuals or groups to prevent the development of infections. In aquaculture, antibiotics at therapeutic levels are frequently administered for short periods of time via the oral route to groups of fish that share tanks or cages. All drugs legally used in aquaculture must be approved by the government agency responsible for veterinary medicine, for example, the Food and Drug Administration (FDA) in the USA. These regulatory agencies may set rules for antibiotic use, including permissible routes of delivery, dose forms, withdrawal times, tolerances, and use by species, including dose rates and limitations. The most common route for the delivery of antibiotics to aquatic animals occurs through mixing the antibiotic with specially formulated feed. However, fish do not effectively metabolize antibiotics and will pass them largely unused back into the environment in feces. It has been estimated that 75% of the antibiotics fed to fish are excreted into the water [22].

Intensive aquaculture has promoted the growth of several bacterial diseases, which has led to an increase in the use of antimicrobials [23, 24]. Current levels of antimicrobial use in aquaculture worldwide are not easy to determine because different countries have different distribution and registration systems and the amount of antibiotics and other compounds used in aquaculture differed significantly between countries [22]. Defoirdt et al. [24] previously estimated that approximately 500–600 metric tons of antibiotics were used in shrimp farm production in Thailand in 1994; they also emphasized the large variation between different countries, with antibiotic use ranging from 1 g per metric ton of production in Norway to 700 g per metric ton in Vietnam.

In most countries with an important aquaculture industry, government agencies exert some controls. For example, in Norway, the use of antimicrobials requires a veterinarian’s prescription, and hence, their use is therapeutic. They are sold in pharmacies or in feed plants authorized by the Norwegian Medicines Agency. In Norway, it is mandatory to report the amount of antibiotics used and retain records of prescriptions.

While other governments, including China, are taking steps to curtail overuse of antibiotics by people, antibiotics in animal feed remain poorly regulated. It has been reported that nearly half of the 210,000 t of antibiotics produced in China are deployed in food animals [25]. Some studies have provided data on what antibiotics are found in waterways and manure in China, which gives an indirect idea of both the amounts and types of antibiotics used. Although based on relatively limited sample sizes, the information available suggests that high volumes of sulphonamides, tetracyclines, and fluoroquinolones (enrofloxacin, fleroxacin, and norfloxacin) are widely used in the Chinese agriculture sector [26–28]. Unauthorized use of pesticides and drugs (malachite green, nitrofurans, fluoroquinolones) has been reported by Chinese farmers in order to increase production and save on the cost of feed. Most fish and shrimp imported from China are cultured in ponds that frequently have poor water quality, and farmers commonly use drugs to control disease and fungal infections. Several imports of fish and shrimps from China to the USA have been detected that violated veterinary drug residue standards indicating evidence of misuse in aquaculture [29].

Overuse is not limited to China. In Nigeria, the administration of veterinary drugs in food animals including aquaculture is characterized by indiscriminate use without appropriate veterinary supervision, regulation, and control to protect consumers. This misuse of veterinary drugs as well as violative residues of antimicrobials in Nigerian livestock and aquaculture has been reported by several authors [16–18, 20, 21].

## Evidence of antimicrobial residues in aquaculture products

In many countries, antimicrobials have been reportedly used indiscriminately in aquaculture [30]. Despite the benefit of improved productivity ascribed to the use of antimicrobials, the risk associated with their residues in the tissues of treated animals or their derived products constitutes health hazards to consumers [31, 32]. Several authors have reported the presence of antimicrobial residues in aquaculture products from different parts of the world. Studies conducted in India [33], Bangladesh [34], Nigeria [35], and Iran [36] have shown evidence of antimicrobial residues in aquaculture products. However, stringent regulations in the USA and the EU have led to high rates of compliance and few reported cases of antimicrobial residues in aquaculture products originating from these countries [37]. Table 3 shows the concentrations of some antimicrobial residues obtained in aquaculture products by country of product origin.

**Table 3:** Documented evidence of antimicrobial residues in aquaculture products

Country	Antibiotic	Concentration (μg kg^−1^)	Product	Reference
India	Erythromycin	41.95	Shrimp	Swapna et al. [33]
Nigeria	Oxytetracycline	553.2	Fish (fillet)	Olatoye and Basiru [35]
Bangladesh	Chloramphenicol	1.91	Shrimp (muscle)	Hassan et al. [34]
Iran	Sulfonamide	7.06	Fish (muscle)	Mahmoudi et al. [36]

Antimicrobial residues are spreading rapidly, irrespective of geographical, economical, or legal differences between countries [38]. A study reported in 2004 by the EU revealed that the majority of residues confirmed in animals were antibacterial agents [7]. Currently, the Joint FAO/WHO Expert Committee on Food Additives (JECFA) has also reported various veterinary drugs and other environmental substance residues in a series of working documents. The JECFA has been participating in further evaluation of the safety of residues of veterinary drugs in food and in establishing ADIs and MRLs for substances when they are administered to food-producing animals in accordance with good veterinary practice [39].

## Legislation concerning veterinary drug use

China as the world’s largest consumer of antimicrobials with loose control of antimicrobial usage is already developing a regulatory framework to deal with the misuse of antibiotics in food-producing animals [40]. This effort is targeted at alleviating the negative impact of antibiotics residues, antimicrobial resistance, and inappropriate use of antibiotics in animal production.

Chinese government regulation began in 2001 with rules and regulations set for approval procedures for the use of veterinary drugs so as to control the use of human medicine for food-producing animals as well as strategies to monitor and control drugs banned for use and measures to control drug residues. However, there is still widespread use of antibiotics in animal feeds as growth promoters in China since animal farming is highly decentralized with small-scale farming accounting for over 70% of the total production, thereby making enforcement and monitoring extremely difficult. So far, the government actions to eliminate the misuse of antibiotics in animals have only been able to reach large-scale producers and antibiotics manufacturers. In contrast to the EU, which imposed a blanket ban on the use of antibiotics as growth promoters as a precautionary measure in 2006, the Chinese government has been trying to seek a compromise over the use and not use of antibiotics in animal feeds for non-therapeutic purposes. In many ways, China’s restrictions are similar to those of the USA. The Chinese regulations allow for the use of antibiotics in food-producing animals for growth promotion and prophylactic purposes, but limit the scope of use within an approved list imposed by the government authorities [41].

In the EU, legislation on residues of veterinary medicines and contaminants is harmonized. The key pieces of legislation are Council Directive 96/23/EC [42] (European Commission, 1996) and Commission Regulation 37/2010/EU [19] (European Commission, 2009). Council Directive 96/23/EC contains guidelines for controlling veterinary drug residues in animals and their products with detailed procedures while the Commission Regulation 37/2010/EU regulates pharmacologically active substances and their classification setting MRLs in foodstuffs of animal origin. The EU has set safe MRLs for these drugs and other veterinary substances, for use as veterinary drugs in animal products entering into the human food chain. The use of veterinary drugs is regulated through EU Council Regulation 2377/90/EC [43], which describes the procedure for establishing MRLs for veterinary medicinal products in foodstuffs of animal origin. MRL values for antibiotics in fish, as set by the EU, are summarized in Table 1.

The Codex Alimentarius Commission, created in 1963 by FAO/WHO, and other regulatory agencies around the world, for example the US Food and Drug Administration (FDA), the Canadian Food Inspection Agency (CFIA), the Australian Pesticides and Veterinary Medicines Authority (APVMA), and the Ministry of Health in Chile, have also set tolerance or MRLS to ensure residues are not present in excess of the set tolerance levels and that no unapproved drugs are used. There are notably differences among MRLs or tolerances set by the different agencies. For instance, only the EU regulation permits the use of fluoroquinolones in fish (Table 1). The Codex Alimentarius Commission and Chilean Ministry of Health have established a MRL for flumequine in trout at 500 μg kg^−1^ while other agencies have set different residue levels for the same drug. The MRL, for the sum of residues of tetracycline in fish, has been set at 100 μg kg^−1^ in the EU countries and Chile, at 200 μg kg^−1^ in Canada and Australia, and at 2000 μg kg^−1^ in the USA.

## Food safety risks associated with aquaculture

Aquaculture products have been associated with certain food safety issues due to the risk of contamination of products by chemical and biological agents. The food safety issues associated with aquaculture vary from region to region and from habitat to habitat as well as according to the method of production, management practices, and environmental conditions. Foodborne parasitic infections, foodborne disease associated with pathogenic bacteria, residues of agro-chemicals, veterinary drugs, and heavy metal contamination have all been identified as potential hazards of aquaculture products [44]. Aquaculture products present the risks of both natural and man-made toxic substances, e.g., microbial, antibiotics, pesticides, and persistent organic pollutants. These contaminants in aquaculture products could pose health concerns to unsuspecting consumers [45]. Extensive use of drugs may increase the risk of an adverse effect of residues on consumers including the development of drug resistance, drug hypersensitivity reaction, disruption of normal intestinal flora, carcinogenic, mutagenic, and teratogenic effects. Considering that nearly 50% of the fish traded in international markets comes from aquaculture, it is important to ensure that the aquaculture sector is producing safe food [46].

Risk analysis has emerged as the basis for assessing, managing, and communicating about risks associated with foodborne hazards. In order to protect public health and facilitate international food trade, the member countries of the WTO have signed the Sanitary and Phytosanitary (SPS) Agreement [46]. With regard to international food safety, standards are set out in the application of the WTO SPS agreement of which risk analysis is a major component. According to the SPS agreement, WTO members have the right to take legitimate measures to protect the life and health of their populations from hazards in food, provided that the measures are not unjustifiably restrictive of trade. Such measures need to be based on risk analysis and take into consideration risk analysis techniques developed by relevant international organizations such as the FAO/WHO Codex Alimentarius Commission. The Code of Practice for Fish and Fishery Products [47] developed by the Codex Committee on Fish and Fishery Products (CCFFP) and the basic texts on food hygiene [48] are the major international documents useful for food safety guidance in aquaculture products.

Biological hazards in fish for human consumption include bacteria, viruses, and parasites. Trematodes are the most important parasites of fish and fish products. There are two broad groups of bacteria of public health significance that contaminate products of aquaculture: those naturally present in the environment indigenous microflora (e.g., *Aeromonas* species, *Clostribuim botulinum*, *Vibrio parahaemolyticus*, *Vibrio cholerea*, and *Listeria* species) and those introduced into the environment by contamination via domestic animal excreta and/or human waste, non-indigenous microflora (e.g., Enterobacteriacae such as *Salmonella* species and *Escherichia coli*).

*Salmonella* and *Vibrio cholerae* found as part of the natural flora of brackish cultured shrimp also pose a major concern for processors and exporters. Many other aquatic pathogens such as several genera *Mycobacterium* and *Vibrio* (especially *M*. *marinum*, *V*. *vulnificus*, and *V*. *parahemolyticus*) as well as species of *Streptococcus*, *Aeromonas*, *Erysipelothrix*, and *Pseudomonas* are known to be contagious to humans, presenting an indication for the use of antimicrobials.

In China, where aquaculture production accounts for nearly 70% of world aquaculture production, a wide variety of chemicals is used by the aquaculture industry, including antibiotics, pesticides, disinfectants, chemotherapeutants, and water conditioners. Although chemical use in Chinese aquaculture is low compared to that used by terrestrial agriculture [49], antibiotic resistance and harm to non-target species are concerns. Many studies reported increases in resistance and even multiple resistances of pathogens as a result of the widespread use of antimicrobials in the Chinese aquaculture [50]. In developed countries, there have been heated debates among stakeholders as to the risks and hazards of the aquaculture system [51]. This does not in any way preclude the importance or significance of aquaculture in the food sector, rather it is a means of resolving issues related to the undesirable effects of the system.

The production system, no doubt, presents risks to public health. Chemicals used in culture fisheries can become disruptive and when they find their way into natural aquatic systems they can cause irreparable damage to the ecosystem. Injuries, preventable occupational diseases, and food safety issues abound in such systems resulting in unnecessary loss of man hours, skilled workforce, and lives [52].

Hazard and risk analysis is a process that provides a flexible framework within which the risks of adverse consequences resulting from a course of action can be evaluated in a systematic, science-based manner [53]. The risk analysis approach permits a defendable decision to be made on whether the risk posed by a particular action or “hazard” is acceptable or not and provides the means to evaluate possible ways to reduce the risk from an unacceptable level to one that is acceptable [54]. The definition of “risk” varies somewhat depending on the sector. Most definitions incorporate the concepts of:

- Uncertainty of outcome (of an action or situation),
- Probability or likelihood (of an unwanted event occurring), and
- Consequence or impact (if the unwanted event happens).

Thus, “risk” is the potential for realization of unwanted, adverse consequences to human life, health, property, or the environment. Its estimation involves both the likelihood (probability) of a negative event occurring as the result of a proposed action and the consequences that will result if it does happen. As an example, taken from pathogen risk analysis, the Aquatic Animal Health Code [55] defines risk as “the likelihood of the occurrence and the likely magnitude of the consequences of an adverse event to public, aquatic animal or terrestrial animal health in the importing country during a specified time period.”

“Risk analysis” is usually defined by either its components and/or its processes. The Society for Risk Analysis [56] offers the following definitions of “risk analysis”:

- A detailed examination including risk assessment, risk evaluation, and risk management alternatives, performed to understand the nature of unwanted, negative consequences to human life, health, property, or the environment;
- An analytical process to provide information regarding undesirable events; and
- The process of quantification of the probabilities and expected consequences for identified risks.

It can also be defined as an objective, systematic, standardized, and defensible method of assessing the likelihood of negative consequences occurring due to a proposed action or activity and the likely magnitude of those consequences, or, simply put, it is “science-based decision-making.” In simple terms, a risk analysis typically seeks to answer four questions:

- What can go wrong?
- How likely is it to go wrong?
- What would be the consequences of its going wrong?
- What can be done to reduce either the likelihood or the consequences of its going wrong? [57–59].

The general framework for risk analysis typically consists of four major components:

- Hazard identification—the process of identifying hazards that could potentially produce consequences;
- Risk assessment—the process of evaluating the likelihood that a potential hazard will be realized and estimating the biological, social, and/or economic consequences of its realization;
- Risk management—the seeking of means to reduce either the likelihood or the consequences of it going wrong; and
- Risk communication—the process by which stakeholders are consulted, information and opinions gathered, and risk analysis results and management measures communicated.

The risk analysis process is quite flexible. Its structure and components will vary considerably depending on the sector. All risk analysis sectors involve the assessment of risk posed by a threat or “hazard.” The definition of “hazard” depends on the sector and the perspective from which risk is viewed, e.g., risks to aquaculture or risks from aquaculture [60]. A hazard thus can be:

- A physical agent having the potential to cause harm, for example:
  - A biological pathogen (pathogen risk analysis);
  - An aquatic organism that is being introduced or transferred (genetic risk analysis, ecological risk analysis, invasive alien species risk analysis);
  - A chemical, heavy metal, or biological contaminant (human health and food safety risk analysis, environmental risk analysis); or
- The inherent capacity or property of a physical agent or situation to cause adverse affects, as in
  - Social risk analysis,
  - Financial risk analysis, and
  - Environmental risk analysis

## Public health implications of antimicrobial use in aquaculture

There has been a surge in the number of foodborne infections caused by antibiotic-resistant bacteria [61]. Recently, a few studies have shown a direct relationship between antibiotic use in food animals and the emergence of antibiotic resistance in human and animal pathogens [62, 63].

Antimicrobials are used in aquaculture as prophylactic or therapeutic measures or as feed additives and also gain access to the pond environment indirectly through the use of human and animal wastes or integrated fish farming system. Human and animal wastes have traditionally been used in Asia as sources of fertilizer for fish culture ponds [64]. The use of waste stabilization ponds is common throughout the world. Moreover, integrated fish farming is also practiced throughout Southeast Asia. Manure from livestock production is administered to fish ponds; the manure is either directly consumed by fish or released as nutrients that support the growth of mainly photosynthetic organisms [65]. This integrated fish farming system produces high yields with low input, with the fish receiving limited, if any, supplementary feed.

Aquatic environments can be a source of resistant bacteria that can be transmitted to and cause infections in humans, and due to the resistance traits result in treatment failures. Such direct spread of resistance from aquatic environments to humans may involve human pathogens such as *Vibrio cholerae*, *Vibrio parahaemolyticus*, *Vibrio vulnificus*, *Shigella* spp., and *Salmonella* spp. or opportunistic pathogens such as *Aeromonas hydrophila*, *Plesiomonas shigelloides*, *Edwardsiella tarda*, *Streptococcus iniae*, and *E*. *coli*. The spread of such resistant bacteria to humans may be through direct contact with water or aquatic organisms, through drinking water, or through the handling or consumption of seafood. Approximately 80% of antimicrobials used in aquaculture enter the environment where they select for bacteria whose resistance arises from mutations or more importantly, from mobile genetic elements containing multiple resistance determinants transmissible to other bacteria. Such selection alters biodiversity in aquatic environments and the normal flora of fish and shellfish. The presence of terrestrial bacteria in aquatic environment together with the presence of residual antimicrobials, biofilms, and high concentrations of bacteriophages with pathogens of human and animal origin can allow exchange of genetic materials between aquatic and terrestrial bacteria. Several recently found genetic elements and resistance determinants for quinolones, tetracyclines, and beta-lactamases are shared between aquatic bacteria, fish pathogens, and human pathogens and appear to have originated in aquatic bacteria [66].

The consequences of antimicrobial resistance in bacteria causing human infections include increased number of infections, frequency of treatment failures and severity of infection, and finally increased costs to society associated with disease. Increased severity of infection includes prolonged duration of illness and increased frequency of bloodstream infections, hospitalization, and mortality [67].

In light of the increased risk posed by antimicrobial resistance, the European Commission (EC) developed an Action Plan (2011–2016), which identified objectives and related measures to be implemented by 2016 to address the problem. The Action Plan took a holistic approach to antimicrobial resistance, focusing on human health and animal health issues, and to a lesser extent, environmental issues. It addressed the problem of antimicrobial resistance at the EU level, including appropriate use of antimicrobials, infection prevention, and the development of new antimicrobials, alternative treatments, and diagnostic tools. The Action Plan also included actions to improve the monitoring and surveillance of antimicrobial resistance and use of antimicrobials in Europe [68].

## Analytical methods for antimicrobial residue detection in aquatic animal tissues

Availability of simple and reliable screening tools for detecting antimicrobial residues in aquatic animal tissues is essential to food safety and consumer protection [69]. Methods for surveillance testing of antimicrobial residues are classified into screening methods and confirmatory methods. While screening methods are used to detect the presence of several analytes including antimicrobial residues in a large number of samples, confirmatory methods such as high-performance liquid chromatography (HPLC) are used to identify and quantify specific antimicrobial residues in samples positive to screening methods [70]. The screening methods usually lack specificity and are qualitative [71]. Methods of analysis of antimicrobial residues present in aquatic animal tissues include microbiological, immunochemical, or physico-chemical methods.

Microbiological (bioassay) methods are based on the principle of bacteria growth inhibition by residues as inhibitors in the test samples. The tests are usually the first-hand method for the analysis of a sample to establish the presence or absence of residues [72] and are used for monitoring and surveillance of antimicrobial residues in large samples of foods of animal origin [73]. Several low-cost, high-sample throughput commercial screening kits have been optimized to prevent false-negative results and have an acceptable number of false-positive results [74, 75].

Immunochemical methods are used to identify specific antimicrobial residue or recognize structurally similar metabolites through the antibody antigen/enzyme interaction [76]. Enzyme-linked immunosorbent assay (ELISA), radioimmunoassay, multi-array, and biosensors have been used for immunochemical screening and/or confirmatory test of residues. Some advanced immunochemical assays can detect less than 10^−9^ mg/dl antimicrobial residue in edible tissues, but are usually expensive [76].

Physico-chemical methods are regarded as confirmatory tests as they detect and quantify antimicrobial residues in edible tissues [77, 78]. The analysis involves extraction of analytes from food matrices, clean-up, detection, and quantification. Examples of physico-chemical methods include HPLC, high-performance thin-layer chromatography (HPTLC), mass spectrometer, and gas chromatography (GC). However, they require expensive equipment and skilled personnel.

Other recent advances in residue analysis include bioluminescent *Escherichia coli* K-12 strain for the detection of the tetracycline family of antimicrobial agents and have been optimized to work with fish samples [79]. Also, chemiluminescence methods comprising micro-flow injection system on a chip have been prepared for the determination of tetracycline in fish and shrimp samples. The method is simple, rapid, and highly sensitive, and reagent consumption is very low.

## Managing antimicrobial residues for food safety

Several international organizations have produced recommendations on the responsible and prudent use of antimicrobial agents in aquaculture to reduce the overuse and misuse of antimicrobials in animals in order to protect public health [80]. Identified basic principles for prudent and rational veterinary use of antimicrobials are as follows.

### Regulation

Regulating the use of antibiotics in food animals is an important part of containing resistance. WHO [81] recommended that national veterinary, agricultural and pharmaceutical authorities, and other stakeholders consider eliminating the use of antibiotics as growth promoters, requiring that antibiotics be administered to animals only when prescribed by a veterinarian, and requiring that antibiotics identified as critically important in human medicine—especially fluoroquinolones and third- and fourth-generation cephalosporins—only be used in food animals when their use is justified.

### Reduced need for and prudent use of antibiotics in aquaculture

Antibiotics are valuable drugs and should be used only therapeutically and as little as necessary. It is important that national veterinary and agricultural and pharmaceutical authorities promote preventive veterinary medicine and the prudent use of antibiotics in collaboration with the private sector and all relevant stakeholders, particularly veterinary practitioners and farmers [81].

Particularly important steps are:

- The need for antibiotics in food animals should be reduced by improving animal health through bio-security measures (to prevent the introduction of harmful bacteria and the development of infections), disease prevention (including the introduction of effective vaccines, prebiotics, and probiotics), and good hygiene and management practices.
- Antibiotics should be administered to food animals only when prescribed by a veterinarian.
- Antibiotics should be used only therapeutically, and the use should be based on the results of resistance surveillance (microbial cultures and antibiotic susceptibility testing), as well as clinical experience.
- Use of antibiotics as growth promoters should be eliminated.
- Narrow-spectrum antibiotics should be the first choice when antibiotic therapy is justified.
- Antibiotics identified as critically important for human medicine—particularly fluoroquinolones and third- and fourth-generation cephalosporins—should only be used in animals if their use is justified.
- The use of antibiotics in food animals should be limited to their approved and intended uses, take into consideration on-farm sampling and testing of isolates from food animals during their production, where appropriate, and include adjustments to treatment when problems become evident.
- International guidelines on prudent use of antibiotics, adapted to countries’ circumstances, should be followed at the national level. Veterinarians’ professional societies should establish guidelines on the appropriate usage of antibiotics for different classes of food animals, including indications of first-, second-, and last-resort choices for treating different bacterial infections.
- Economic incentives that facilitate the inappropriate prescription of antibiotics should be eliminated.

Prudent veterinary use of antimicrobials is championed in the UK by the Responsible Use of Medicines in Agriculture Alliance, an alliance which produces a variety of resources aimed at disseminating good veterinary and antimicrobial chemotherapy agriculture practices. In addition, the British Veterinary Association (BVA) and the British Small Animal Veterinary Association (BSAVA) produce resources outlining responsible veterinary practice. The Department for Environment, Food and Rural Affairs is involved in the surveillance of veterinary medicine, which provides a useful resource for monitoring antimicrobial use in this sector [82].

### Surveillance

The surveillance of antibiotic resistance in zoonotic and commensal bacteria in different food animal reservoirs and aquaculture products is a prerequisite for understanding the development and dissemination of antibiotic resistance, providing relevant risk assessment data, and implementing and evaluating targeted interventions. This surveillance entails specific and continuous data collection, analysis, and reporting that quantitatively monitor temporal trends in the occurrence and distribution of resistance to antibiotics. To monitor trends in resistance and allow for timely corrective action and evaluation of interventions, it is suggested that public health, veterinary, and food authorities consider:

- Establishing a surveillance system for the usage of antibiotics in people and food animals and
- Establishing an integrated (among the public health, food, and veterinary sectors) surveillance system to monitor antibiotic resistance in selected foodborne bacteria [83].

The EU Commission, in order to manage the public health risk of antimicrobial resistance and to evaluate the impact of interventions, relies on European Food Safety Authority (EFSA), European Centre for Disease Prevention and Control (ECDC), and European Medicines Agency (EMA) work and supports wide-ranging and well-validated surveillance networks on antimicrobial residue and antimicrobial consumption for both humans and animals as part of the European Surveillance System (TESSy). In Europe, sales of veterinary antimicrobial agents have been monitored since 2010 through the European Surveillance of Veterinary Antimicrobial Consumption (ESVAC) [84].

### Advocacy and communication

The main objectives of advocacy and communication on antimicrobial residues at the international and national levels should be to raise awareness of the importance of antibiotics in treating bacterial infections and the public health challenges of antimicrobial residues—including within a food safety perspective—and to prompt action to use them prudently in all sectors. A participatory approach should be used to develop and implement communication strategies that emphasize the importance and benefits of prudent use principles. These strategies should identify relevant target audiences, such as decision-makers; professionals from the health, veterinary, and agricultural sectors; farmers; the media; and the general public. These audiences need trustworthy and evidence-based information to guide their decisions and choices [81].

### Training and capacity building

Education strategies that emphasize the importance and benefits of the prudent use of antibiotics should be developed and implemented to provide relevant information about antibiotic resistance to farmers, veterinarians, and the public. There is an urgent need to develop guidelines on prudent use, with multidisciplinary involvement, to reduce misuse of antibiotics in aquaculture, giving special consideration to antibiotics categorized as critical for human medicine. Veterinarians and farmers should receive training in following these guidelines and, to improve compliance, need to be audited and to receive feedback [81].

### Knowledge gaps and research needs

The understanding of antibiotic resistance related to food safety still has many knowledge gaps that research is needed to fill. For example, the available information on the burden of disease from antibiotic-resistant organisms is mainly qualitative; research to quantify the differential burden of disease that results from resistant versus susceptible bacterial strains needs to be promoted. Such information would provide an additional dimension to the magnitude of the issues and support risk assessment and management, including the development of cost-effective strategies to counteract the development and spread of antibiotic resistance [81].

### Hazard analysis of critical control points (HACCP)

The HACCP system, which is science-based and systematic, identifies not only specific hazards but also measures for their control to ensure the safety of food. HACCP is a tool used to assess hazards and establish control systems that focus on prevention rather than relying mainly on end product testing. HACCP can be applied through the food chain from primary production to final consumption, and its implementation should be guided by scientific evidence of risks to human health. In addition, the application of HACCP systems can aid control by regulatory authorities and promote international trade by increasing confidence in safety of traded foods [85].

According to published information, HACCP systems are being put into practice in aquaculture at various levels but mainly in the sectors of high-valued farmed species such as salmon in Norway, Canada, Ireland, USA, New Zealand, UK, and Chile; shrimp in Thailand, Ecuador, Australia, Cuba, Brazil, Central American countries, and USA; trout in European countries, Argentina, Peru, and Brazil; catfish in USA; crawfish in USA; and bullfrogs in Brazil. The USA has the largest volume of information and guidelines on how to apply HACCP in aquaculture with catfish farming receiving the major coverage [86, 87].

In the case of developing countries, the application of the HACCP concept in aquaculture is mainly influenced by the need to comply with sanitary requirements of the main importing countries. Serious restrictions imposed by Japan on the importation of farmed shrimp contaminated with residues of veterinary drugs, particularly antibiotics, forced government and producers/exporters in Thailand, Indonesia, and the Philippines to implement the new control systems.

While many agriculture experts believe that the application of HACCP may be difficult at the “farm level,” a number of authors have considered that the application of HACCP in aquaculture is suitable [88–92]. HACCP was considered a superior method of fish inspection by the participants of the International Conference on Quality Assurance in the Fish Industry held in Lyngby, Denmark, in 1991. Participants agreed that the HACCP concept should be applied in the fish industry to cover food safety, plant/food hygiene, and economic fraud issues [86]. During the Second International Conference on Fish Inspection and Quality Control held in Washington, DC, USA, in 1996, participants affirmed that HACCP-based programs were in the process of being implemented on a global scale. Governments and industry alike were urged by this International Conference to continue their efforts and to give a high priority to the full implementation of HACCP-based systems [93].

## Conclusion

Aquaculture production has become an increasingly important means of producing aquatic products for the human consumption. The use of antimicrobials in aquaculture results in deposition of residues in edible portions of fish and other aquaculture products. While low level residues of certain antibiotics are considered safe in some food products, residues of other antibiotics (e.g., chloramphenicol) may pose an unacceptable risk to public health and are therefore prohibited for use in food animals.

The responsibility for food safety associated with aquaculture products is shared among governments, the fish production and processing industries, and consumers. As pointed out by the World Organisation for Animal Health (OIE), antibiotics are essential tools for protecting animal health and animal welfare [94]. Leaving sick food animals untreated poses a risk to both food safety and public health as more than 60% of human pathogens today originate from animals [94]. When used appropriately, antibiotics also contribute to satisfying the increasing world demand for safe food of animal origin such as fish. Without antibiotics, food animals suffering from bacterial infectious diseases will be denied effective treatment and outbreaks of disease may not be effectively controlled or prevented within a herd.

The OIE, WHO, and FAO all recommend prudent and responsible use of antimicrobials including antibiotics [94]. In general, antibiotics should only be used when indicated and when used in food animals, they should be under veterinary supervision. Aquaculture products should be screened for safety levels of antimicrobial residues. Efforts should also be made to reduce the use of antibiotics by implementing good animal husbandry practices.

Several alternatives to antibiotics have been developed, including probiotics, phage therapy, and essential oils, and some of these have been successfully used to control bacterial infections in aquaculture facilities [95]. These microorganisms, compounds, and/or their components are gaining increasing interest because of their relatively safe status, wide acceptance by consumers, and their potential for multipurpose uses. Although the application of these alternatives to aquaculture is very promising, further studies are needed to gain more insight about their mechanisms of actions, to improve their stability and to evaluate their impact on the environment and the host microbiota.

Conclusively, there is a need for field surveys and improved record keeping of antimicrobial sales on a country and species basis for establishing comprehensive antimicrobial use databases for aquaculture. Such databases should be used to identify global hotspots in which antimicrobials are disproportionally used and that require urgent attention and better management. Also, risk assessment approaches for preventing diseases and the development and spread of antimicrobial resistance bacteria in aquatic environments need to be established. Identifying the two-way link between antimicrobial use in aquaculture and antimicrobial resistance in humans is also of critical importance as the aquatic environment often constitutes the final receptacle of both anthropogenic and livestock waste.

## Recommendation

There can be no doubt that antimicrobial resistance poses a global challenge. No single nation, however effective it is at containing resistance within its boundaries, can protect itself from the importation of resistant pathogens through travel and trade. The global nature of resistance calls for a global response, not only in the geographic sense, i.e., across national boundaries, but also across the whole range of sectors involved. Nobody is exempted from the problem, nor from playing a role in the solution. Therefore, relevant recommendations must be targeted to the following stakeholders:

- Policy-makers and health authorities
- Industry
- Research

### Policy-makers and health authorities

The impact of antimicrobial resistance on animals and humans cannot be overemphasized. For animal health, the main issue is treatment failure due to increases in resistance. For human health, the main concern is adverse health effects associated with the presence of residues in the food produced or resistance in bacteria associated with human disease. Resistance in bacteria causing human disease may arise either directly via enrichment of these bacteria in the aquaculture environment or indirectly via enrichment of the genes that encode such resistance and which may subsequently be transferred to bacteria associated with human disease. Considering the principle of One Health, policy-makers and health authorities should integrate efforts that embrace human and veterinary disciplines in a holistic pattern. They must appreciate the need to:

- Embrace bio-security standards and vaccination plans as preventive measures in human and animal practice to limit the need of antimicrobials use;
- Generate relevant data to assess the economic and health impacts of antimicrobial resistance;
- Ensure early detection of risks, at a global level (European Commission proposal for a Regulation on animal health creates a legal basis for the appropriate surveillance and early detection of listed pathogens in animals, among those, potentially, antimicrobial resistant ones [84] by rapid diagnostics that accelerate the identification and treatment of resistant pathogens);
- Develop a system for collecting data deriving from official controls carried out by public veterinarians; and
- Improve the comparability of data on resistance and use of antimicrobials both in human and veterinary medicine for strengthening risk management decisions and properly evaluating the measures taken.

### Industry

National governments have an important legislative role in ensuring the appropriate manufacture, licensure, and sale of antimicrobials and also an important responsibility in ensuring that these drugs are promoted in a fair and accurate manner. Government controls on drug promotional activities and compliance of the pharmaceutical industry with both legislation and agreed codes of practice are important factors if appropriate antimicrobial use is to be encouraged. Recommendations for intervention are:

- Introduction of requirements for pharmaceutical companies to comply with national or international codes of practice on promotional activities;
- Ensure that national or international codes of practice cover direct-to-consumer advertising, including advertising on the Internet;
- Institute systems for monitoring compliance with legislation on promotional activities;
- Identify and eliminate economic incentives that encourage inappropriate antimicrobial use; and
- Raise prescriber awareness that promotion in accordance with the datasheet may not necessarily constitute appropriate antimicrobial use.

### Research

Scientific research and innovation serves as a basis for science-based policy and legal measures to address antimicrobial resistance and can provide new tools for diagnosis and treatment. Diagnostic tools that include tests for quick and accurate identification of pathogenic microorganisms and/or for determining their sensitivity to antimicrobials play a key role in the fight against microbial infections. Vaccines and other preventive measures could have an important impact on reducing the spread of infections and, thus, the need for treatment. Therefore, research and innovation in these fields should be supported. Possible recommendations for intervention are:

- Encouraging cooperation between industry, government bodies, and academic institutions in the search for new drugs and vaccines;
- Encouraging drug development programs that seek to optimize treatment regimens with regard to safety, efficacy, and the risk of selecting resistant organisms;
- Promoting further research aiming to better understand antimicrobial resistance and pathogenic-host interactions;
- Promoting further research on the development of diagnostic tools, vaccines, and other preventive measures; and
- Contributing to a global mapping of drug resistance.

## Abbreviations

ADI: Acceptable daily intake
APVMA: Australian Pesticides and Veterinary Medicines Authority
ARB: Antibiotic-resistant bacteria
BSAVA: British Small Animal Veterinary Association
BVA: British Veterinary Association
CCFFP: Codex Committee on Fish and Fishery Products
CCPs: Critical control points
CFIA: Canadian Food Inspection Agency
EAAD: European Antibiotics Awareness Day
ECDC: European Centre for Disease Prevention and Control
EFSA: European Food Safety Authority
ELISA: Enzyme-linked immunosorbent assay
EMA: European Medicines Agency
ESVAC: European Surveillance of Veterinary Antimicrobial Consumption
FAO: Food and Agriculture Organization
FDA: Food and Drug Administration
HACCP: Hazard Analysis and Critical Control Points
HPLC: High-performance liquid chromatography
HPTLC: High-performance thin-layer chromatography
LAST: Live Animal Swab Test
MRLs: Maximum residue limits
NOEL: No Observable Effect Level
OIE: World Organization for Animal Health
PAHs: Polycyclic Aromatic Hydrocarbons
PPB: Parts per billion
PPT: Parts per trillion
STOP: Swab Test On Premises
WHO: World Health Organization
WTO: World Trade Organization
